# Patterns of Extrafloral Nectar Production in *Chamaecrista fasciculata* (Fabaceae: Caesalpinoideae)

**DOI:** 10.17912/micropub.biology.001534

**Published:** 2025-06-26

**Authors:** Madeline C. Marquardt, Jordan L. Reed, Susana M. Wadgymar

**Affiliations:** 1 Davidson College, Davidson, North Carolina, United States

## Abstract

*Chamaecrista fasciculata* develops extrafloral nectaries at the base of most of its leaves that attract a variety of insects, including ants that aid in defense against herbivores. Here, we show that the extrafloral nectaries on newly developed leaves are larger and produce more nectar than those on older leaves. In addition, we demonstrate that nectar production does not increase with regular nectar removal, as might be experienced with routine visitation by patrolling ants, suggesting that the mass of nectar produced by individual nectaries is not plastic in response to removal. It’s possible that plants prioritize producing nectar closer to their apical meristems to encourage ants to patrol and defend the full extent of their vegetative structures or to protect the tissues most vulnerable to herbivory.

**Figure 1. Patterns of extrafloral nectar production:**
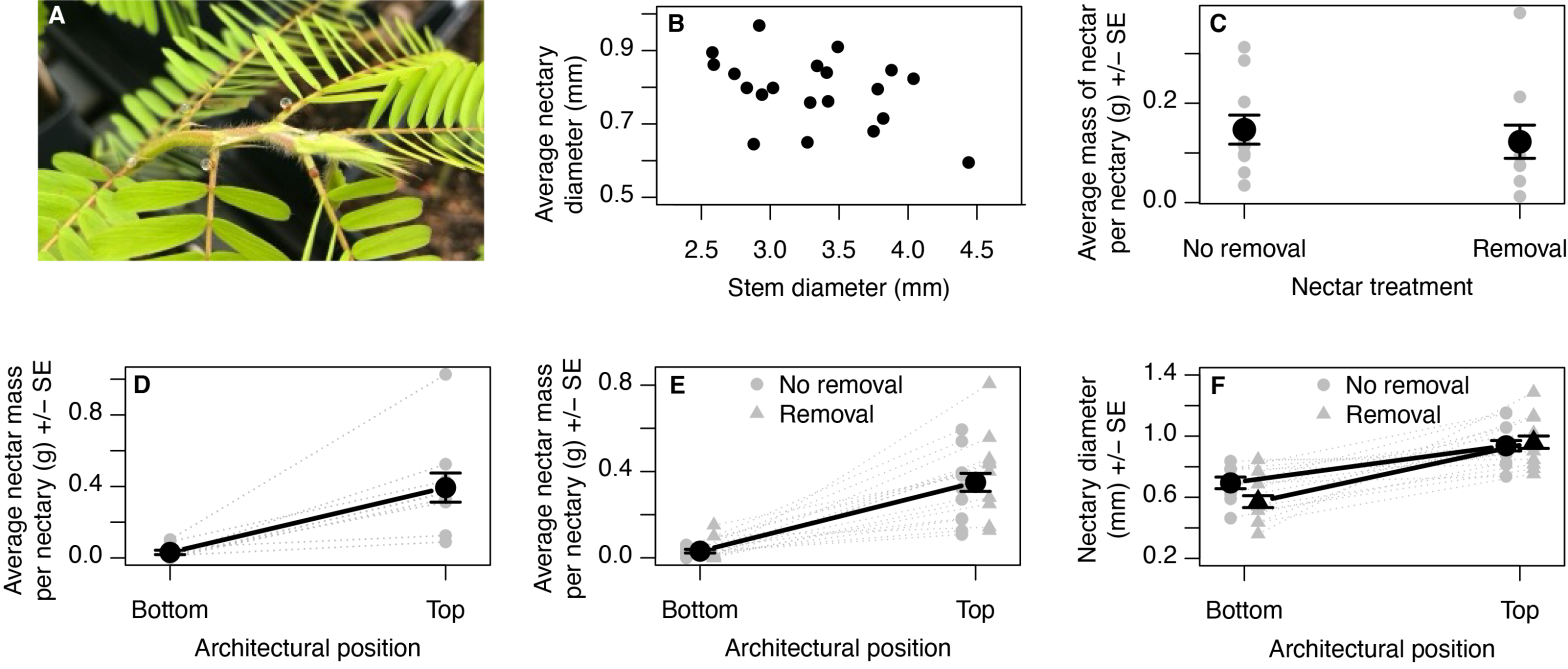
A) The top portion of a *Chamaecrista fasciculata* branch. Extrafloral nectaries develop at the base of each leaf and can be seen here with several days worth of nectar accumulated on them. B) A scatterplot of stem diameter (mm) against the plant-wide average diameter of extrafloral nectaries. C) The mass of nectar produced per extrafloral nectary of plants from the first observation period in the removal treatment versus the control treatment where nectar was collected from all nectaries. D) The average mass of nectar produced during the second observation period by the lowest (oldest) three versus the highest (newest) three extrafloral nectaries. E) The average mass of nectar produced from the bottom and top three extrafloral nectaries for plants from the third observation period. F) The average size of nectaries from the bottom and top three extrafloral nectaries for plants from the third observation period. In panels c through f, grey dots represent values for individual plants while the larger black circle and error bars reflect the average +/- the standard error for the variables listed on the x-axis.

## Description

Extrafloral nectaries are nectar-secreting glands on plants that develop on non-floral tissues such as leaves and petioles. They are estimated to have evolved independently at least 450 times, with species that produce extrafloral nectaries distributed across approximately 21% of plant families (Weber and Keeler 2013). Extrafloral nectaries contribute indirectly to a plant defense by attracting invertebrates that act as predators of, or guards against, herbivores (Weber and Keeler 2013). In response to variation in herbivory, plasticity has been observed in extrafloral nectary traits including nectar nutrition and volume. For instance, the simulation of herbivory via fruit and flower bud cutting on *Pachycereus schotti* and leaf cutting on *Qualea multiflora* increased the production of nectar by extrafloral nectaries (Holland et al. 2009; Calixto et al. 2021). Similarly, extrafloral nectar production increased in *Brassica juncea* when exposed to certain herbivore species (Mathur et al. 2013). Nectar production may be energetically costly (Pyke and Ren 2023), as evidenced by reduced nectar production in populations that experience low levels of herbivore damage (Rios et al. 2008). Thus, herbivore-induced plasticity in extrafloral nectary traits is likely an adaptive defense strategy for the species that produce them. Here, we ask whether the mass of nectar secreted by extrafloral nectaries varies between older versus newer nectaries and is plastic in response to nectar removal.

*Chamaecrista fasciculata* (Michx.) Greene (Fabaceae), or the partridge pea, produces extrafloral nectaries on the petioles of its pinnately compound leaves (Figure 1a). Nectaries typically first appear on the fourth true leaf to develop and on every leaf thereafter, with greatest nectar production from those on newly mature leaves (Rios et al. 2008). The extrafloral nectaries of *C. fasciculata* provide indirect defense against herbivores by attracting ants that regularly patrol the plants and guard the nectaries (Rutter and Rausher 2004). To illustrate, leaf damage by herbivores was found to double in treatments where ants are excluded (Rios et al. 2008). Growth is indeterminate, with leaves, flowers, and fruit developing continuously so long as conditions are favorable and resources are available. Flower buds develop from racemes at each leaf node, potentially making nearby extrafloral nectaries a crucial component of defense against florivores and frugivores. For instance, *C. fasciculata* plants suffered higher fruit predation and had lower reproductive output at sites where the abundance of ants and potential herbivores was absent (Barton 1986). Plasticity in nectar production may be advantageous given that *C. fasciculata* populations can experience extensive variation in ant and herbivore abundance (Barton 1986; Rios et al. 2008). We hypothesize that plants will increase nectar production when experiencing regular nectar removal, as they might encounter with routine visitation by patrolling ants.

We found the average size of extrafloral nectaries within a plant were negatively correlated with stem diameter (r=-0.407, Fig. 1b) and total leaf number (-0.206), such that larger plants tended to have smaller nectaries. Plants were randomly assigned to nectar removal treatment or control treatment without regard to their size. During the first observation period, we observed no difference in the mass of nectar produced in 24-hours between non-reproductive plants that experienced seven consecutive days of nectar removal from all extrafloral nectaries versus those that did not (Fig. 1c; F _1,17_ =0.288, p=0.599). We included total leaf number as a covariate and note that it also did not explain variation in the average mass of nectar produced per nectary (F _1,17_ =0.369, p=0.552). It is possible that the amount of extrafloral nectar produced is not plastic in response to removal, or perhaps that plasticity is elicited by other factors not manipulated here, such as herbivore damage. Alternatively, our study was conducted on non-reproductive plants, and patterns of extrafloral nectar production may change as plants progress through ontogeny (Holland et al. 2009).

Extrafloral nectaries found on newly developed leaves produced more nectar than those on older leaves during the second observation period (Fig. 1d; F _1,9_ =23.171, p=0.001). A third observation period confirmed that this pattern was not affected by artificial nectar removal treatments (Fig. 1e; Nectary position*Treatment F _1,18_ =0.0865, p=0.365). Greater nectar production on newly produced leaves has been observed across taxonomically diverse species (e.g., Heil et al. 2000; Jones and Koptur 2015). Plants may prioritize producing nectar closer to their apical meristems to encourage ants to patrol and defend the full extent of their vegetative structures or to protect the tissues most vulnerable to herbivory. Extrafloral nectaries are larger on newer leaves (Fig. 1f), which may explain why they produce more nectar. Alternatively, nectaries on older leaves may decline in function or size over time, which would necessitate a longer-term monitoring effort than we present here.

Nectaries on older leaves that experienced nectar removal were marginally smaller than those on plants that did not have nectar removed, but this treatment effect was not observed on newer nectaries (Fig. 1f; Nectary position*Treatment F _1,98_ =3.860, p=0.052). Despite the care we took when handling plants, it is possible that nectaries experiencing regular nectar removal were damaged or stunted by having the nectar gently wiped off with tissue. However, we note that this technique has been successfully used by pollination biologists for decades (Kearns and Inouye 1993), and filter paper has previously been used to collect nectar in *C. fasciculata* (Rios et al. 2008). In addition, the range of nectary diameters in both treatments were very similar (Fig. 1f), emphasizing that the marginal difference in nectary sizes between treatments may not be biologically significant.

In sum, we found that larger plants produce smaller nectaries, on average, and that plants produce more nectar from nectaries on newly developed leaves. We also demonstrate that nectar removal does not induce increased nectar production, aligning with previous studies on nectar induction in *C. fasciculata* (Rios et al. 2008). To fully characterize extrafloral nectar plasticity and fitness effects, future investigations could examine extrafloral nectar production throughout ontogeny (Villamil et al. 2013) and in the context of different types of herbivory (e.g., leaf, flower, and fruit removal treatments). It would also be valuable to compare the results acquired through different nectar collection methods including microcapillary tubes, syringes, and centrifugation (Kearns and Inouye 1993). Given the proximity of extrafloral nectaries to reproductive tissues and indeterminate growth and flowering, we propose that *C. fasciculata* is an excellent model for plant biologists exploring optimal defense theory or for myrmecologists studying optimal foraging theory.

## Methods

*Study design and data collection*

*Chamaecrista fasciculata* seeds were ordered in 2023 from Prairie Moon Co. (Winona, MN, USA). Twenty healthy, non-flowering plants were reared in a climate-controlled greenhouse and were randomly assigned to either a control treatment or a nectar removal treatment. Plants were manually watered each day until the soil within the pots was saturated and we made every effort to ensure that plants were watered consistently. Extrafloral nectar on plants in the nectar removal treatment was gently removed via absorption by Kimwipes (Kimberly-Clark Co, USA).

*First observation period:* All nectaries on a given plant were cleared every 24 hours for seven consecutive days, with analyses conducted on the nectar collected on the seventh day. Plants in the control treatment experienced no nectar removal until the sixth day, at which point nectar was removed from all nectaries to allow for 24 hours of nectar replenishment before data collection. On the seventh day, after all plants had 24 hours to replenish their nectaries, nectar from each nectary was absorbed into pre-weighed pieces of Kimwipe and were weighed immediately afterward on a microbalance (Mettler Toledo MT5 Micro Analytical Balance). The total mass of nectar was measured as the difference in the mass of the Kimwipe before and after nectar removal.

*Second observation period:* As a follow-up observation several days later, we collected and separately weighed nectar from the top three and the bottom three nectaries to evaluate variation in nectar production across plant architecture.

*Third observation period:* To evaluate whether the nectar removal treatment had distinct effects on lower versus upper nectaries, we collected nectar in a third observation period as described above, with nectar collected from lower three and upper three nectaries after plants experienced nectar removal (or not) for seven days. We used calipers to measure nectary diameter for the top three and bottom three nectaries and to measure each plant’s stem diameter at soil level. We also counted the number of leaves on all plants.

*Statistical Analysis*

For data from the first observation period, we assessed whether nectar removal induced greater nectar production using a linear regression with Treatment as a fixed effect and Total Leaf Number as a covariate. To determine whether older versus younger nectaries produced different amounts of nectar during the second observation period, we used a linear mixed model with Position as a fixed effect and Plant ID as a random effect. We analyzed nectar mass and nectary size data from the third observation period using mixed models, with Position, Treatment, and their interaction as fixed effects and Plant ID as a random effect. Residuals were visually inspected for normality and heteroskedasticity. The significance of explanatory variables was determined using log likelihood tests. All analyses were conducted in R (R Core Team 2021) and mixed models used the nlme package (Pinheiro et al. 2017).
